# Inhibition of Circular RNA 006029 Alleviates Pancreatic β-Cell Injury Through the AKT/mTOR Signaling Pathway

**DOI:** 10.2174/0109298673401334251201092718

**Published:** 2026-02-04

**Authors:** Zhen Wang, Zheli Luo, Li Wang, Jiaxing Feng, Kunlin Huang, Ying Zheng, Zhiguang Zhou

**Affiliations:** 1 Department of Metabolism and Endocrinology, Guilin Hospital of the Second Xiangya Hospital CSU, Guilin, 541100, China;; 2 Key Laboratory of Diabetes Immunology, Department of Metabolism and Endocrinology, National Clinical Research Center for Endocrine and Metabolic Diseases, Ministry of Education, The Second Xiangya Hospital of Central South University, Changsha, 410011, China;; 3 Department of Endocrinology, Shenzhen Second People’s Hospital, First Affiliated Hospital of Shenzhen University, Shenzhen, 518000, China;; 4 Department of Thoracic Surgery, The Second Xiangya Hospital, Central South University, Changsha, Hunan, 410011, China;; 5 Center for Medical Research, The Second Xiangya Hospital, Central South University, Changsha, Hunan, 410011, China

**Keywords:** Type 1 diabetes, pancreatic β-cell injury, circular RNA 006029, AKT, mTOR, Rapamycin

## Abstract

**Introduction:**

Type 1 Diabetes Mellitus (T1DM) is characterized by the damage of pancreatic β-cells induced by autoimmune responses. Circular RNAs (circRNAs) play important regulatory roles in the pathogenesis of T1DM, but the underlying mechanisms require further substantiation.

**Methods:**

This study focused on a novel circRNA, circ006029, to investigate its regulation on β-cell damage. The potential involvement of circ006029 in β-cell proliferation, apoptosis, autophagy, and inflammatory responses was investigated using CCK-8, qRT-PCR, and immunoblot assays. The utilization of a cytokine mixture, and specific molecular blockers Rapamycin and Capivasertib, was applied to investigate the pathway by which circ006029 regulates β-cell damage. Transcriptome sequencing and bioinformatics analysis were conducted to explore differentially expressed mRNAs related to circ006029 regulation.

**Results:**

The expression of circ006029 was observed to increase in damaged MIN6 cells. The inhibition of circ006029 serves a protective role in MIN6 β-cells by promoting β- cell proliferation and attenuating apoptosis. circ006029-knockdown could augment β- cell autophagy and attenuate apoptosis through the AKT/mTOR signaling pathway. Moreover, circ006029 might be involved in the inflammatory response of MIN6 cells.

**Discussion:**

The knockdown of circ006029 was demonstrated to alleviate β-cell inflammation and reduce cell apoptosis. The promotion of β-cell proliferation and heightened autophagy also substantiated the protective effects of circ006029 silence. Furthermore, we also proved that circ006029 might contribute to autophagy *via* the AKT/mTOR signaling pathway. All the results implied that the presence of circ006029 may drive a detrimental regulatory role in pancreatic β-cells. This may provide valuable evidence that circ006029 might be a potential target for alleviating β-cell damage in T1DM and rebuilding β-cell function.

**Conclusion:**

These findings suggest that circ006029 may serve a detrimental role in β- cell damage, which provides new ideas for exploring the mechanism of β-cell damage in early insulitis in T1DM.

## 1 INTRODUCTION

Type 1 Diabetes Mellitus (T1DM) has been driving scientists’ attention for decades, especially since the 1970s when *Bottazzo, G F *et al.** and *MacCuish, A C *et al.** successively proved that the presence of circulating islet cell-related antibodies is an important mechanism of the kind of insulin-dependent diabetes [1, 2]. Increasing evidence indicates that the most fundamental trigger of T1DM is an imbalance in the immune system, leading to severe insulin deficiency due to the loss of insulin-producing β-cells. As is well known, autoreactive T cells can act as the primary effector cells in inducing β-cell apoptosis [3]. In addition, islet-infiltrating immune cells could secrete many types of pro-inflammatory factors, including cytokines such as IFN-γ, IL-1β, TNF-α, and IL-6, and chemokines such as IL-8, Monocyte Chemoattractant Protein-1 (MCP-1), and C-C Motif Chemokine Ligand 5 (CCL5) [3, 4], which could cause an irreversible damage of pancreatic β-cells, ultimately leading to absolute inadequate insulin secretion and β-cell apoptosis. Autophagy is an important endogenous mechanism of reducing ROS and protecting β-cells against apoptosis [5]. Pancreatic β-cell autophagy has been reported to be impaired in T1DM, which contributes to β-cell apoptosis in T1DM [6]. However, the specific regulatory mechanisms of pancreatic β-cell autophagy dysfunction in T1DM have not been clarified yet.

Non-coding RNAs (ncRNAs) have been reported to show significant regulatory roles in T1DM, especially in the inflammatory process. Most of which were long non-coding RNAs (lncRNAs), microRNAs (miRNAs), *etc.* *Motterle, Anna *et al.** reported significant modulation of lncRNAs *via* distinct inflammatory pathways in injured β-cells in NOD mice with insulitis [7]. From clinical patient specimens, lncRNA SRA was substantiated to act as an miR-146b sponge, closely associated with metabolic reprogramming and β-cell apoptosis [8]. Dysregulation of plasma-derived exosomal lncRNAs has been shown to favor β-cell injury and contribute to T1DM progression [9]. *Roggli E *et al.** reported that the miR-29 family is a pivotal promoter of βcell death by downregulating the antiapoptotic protein Mcl1 [10]. Interestingly, extrinsic miRNAs such as miR-142-3p/-5p and miR-155, which were transported from T lymphocyte-derived exosomes, were also found to favor the loss of β-cells [11]. Our previous findings also showed that miR-101a and miR-30b not only affect insulin production but also trigger β-cell failure upon overexpression of these miRNAs [12].

Circular RNAs (circRNAs) are a class of ncRNAs characterized by a covalently closed single-stranded RNA loop with no 5’ and 3’ ends. Research on the interplay between circRNAs and β-cell damage has been shown during the past few years. circHIPK3 and ciRS-7/CDR1as have been demonstrated to have protective effects on insulin-secreting cells by preventing apoptosis [13]. Monocyte-specific circPPM1F was verified to be involved in MΦ activation, and the overexpression of circPPM1F exacerbated β-cell injury [14]. Our previous research demonstrated that prolonged exposure to inflammatory conditions led to β-cell failure and the dysregulation of circRNAs, including circRNA 006029 (upregulated), circRNA 000286 (downregulated), and circRNA 017277 (downregulated) *in vitro*, suggesting a potentially crucial role of circRNAs in β-cell injury during the progression of T1DM [15].

Herein, we primarily investigated the potential regulatory mechanism of circRNA 006029 in cytokine-induced β-cell injury. We demonstrated that circ006029 is involved in pancreatic β-cell injury by regulating the AKT/mTOR signaling pathway [16].

## 2 MATERIALS AND METHODS

### 2.1 Cell Culture

The murine insulin-secreting cell line MIN6 was originally purchased from ATCC and cultured in Dulbecco’s modified Eagle’s medium (DMEM, Gibco Laboratories, Grand Island, NY, USA), supplemented with 15% Fetal Bovine Serum (FBS, Bovogen, Australia) and 70 mmol/L β-mercaptoethanol (Genview, China). Cells were cultured at 37°C with a humidified atmosphere of 5% CO_2_. To simulate the microenvironment of min6 cells in the early stage of type 1 diabetes inflammation and construct a cell damage model, cells were seeded (1 x 10^6^ per well, 6-well plate) and exposed to cytokine cocktails consisting of 25 ng/ml IFN-γ (PeproTech, USA), 2.5 ng/ml IL-1β (PeproTech, USA), and 2.5 ng/ml TNF-α (PeproTech, USA) for 24 hours [17].

### 2.2 Cell Transfection

For inhibition of circRNA 006029 (circ006029), cells were seeded (1 × 10^6^ per well, 6-well plate) one day before transfection with circ006029 siRNA (Sequences in Supplementary Table **1**) and the corresponding negative control (NC), which were obtained from GenePharma (China). The transfection was performed with Lipofectamine™ RNAiMAX (Invitrogen, CA, USA) according to the manufacturer’s protocol.

### 2.3 RNA Isolation and Quantitative Real-Time PCR

Total RNA was extracted from MIN6 cells using TRIzol reagent (Life Technologies, USA) and was quantified by using Nanodrop (ThermoScientific, USA), followed by reverse transcription of RNA into cDNA using *Evo M-MLV* RT Kit with gDNA Clean for qPCR II (Accurate Biology, China) following the manufacturer’s instructions. The qPCR was performed on a Bio-Rad CFX ConnectTM Real-Time System using the SYBR Green Premix *Pro Taq* HS qPCR Kit (Accurate Biology, China). Reactions were performed in triplicate in a 20 μL PCR reaction in the following steps: Denaturation at 95°C for 3 minutes, followed by 40 cycles of amplification at 95°C for 5 s and annealing and extension at 60°C for 30 s. U6 and β-actin were used as internal controls. Results were determined by using the 2^−ΔΔCT^ method (Sequences in Supplementary Table **2**).

### 2.4 Cell Counting Kit (CCK-8) Assay

The effects of circ006029 on MIN6 cell proliferation were determined by Cell Counting Kit-8 (CCK-8) (Abiowell, China) in accordance with the manufacturer’s protocol. MIN6 cells (1.7 × 10^3^ per well) were seeded into 96-well plates. After transfection of circ006029 or NC siRNA, the cells were cultured for an additional 12 hours, 24 hours, and 48 hours, respectively. The CCK-8 reagent was added into each well, and the plate was incubated at 37°C for 2 hours. After incubation, the absorbance at 562 nm was measured using a Varioskan LUX (Thermo Scientific, USA).

### 2.5 Subcellular Hierarchical Analysis

Subcellular separation of MIN6 cells was conducted by using the Cytoplasmic and Nuclear RNA Purification Kit (Norgen, Belmont, CA, USA) prior to the reverse transcription process, following the manufacturer’s instructions. β-actin and U6 were used as internal references for cytoplasmic and nuclear RNA, respectively.

### 2.6 CircRNA Stability Determination

The total RNA of MIN6 cells was extracted using TRIzol, and then digested with RNase R using a kit obtained from Yeasen Biotechnology (China), at a concentration of 1 U per 3 μg of RNA at 37°C for 5 minutes, followed by an enzyme deactivation step at 70°C for 10 minutes. Besides, MIN6 cells were incubated with 5 μg/ml of Actinomycin D (Aladdin, China) for 2, 4, 6, and 12 hours to inhibit RNA transcription. Then, qRT-PCR was performed as described in the RNA isolation and Quantitative Real-Time PCR section to validate the characteristics of circ006029.

### 2.7 Flow Cytometry

Apoptosis analysis of MIN6 cells was performed by flow cytometry using the Annexin V-FITC/PI Apoptosis Assay Kit (Biosharp, China) according to the manufacturer’s instructions. Briefly, MIN6 cells were transfected with circ006029 or NC siRNA. MIN6 cells in the culture medium and the adherent cells were both harvested. After being gently washed and resuspended with phosphate buffer solution (PBS, BasalMedia, China), cells were stained with an appropriate amount of Annexin V and PI in the dark. Apoptosis was measured on a CytoFLEX SRT (Beckman Coulter, USA), and the percentage of apoptotic cells was analyzed in FlowJo.

### 2.8 The Luciferase Reporter Assay

MIN6 cells were seeded (1 × 10^6^ per well, 6-well plate) and transfected with circ006029 or NC siRNA along with NF-*κ*B activity luciferase reporter plasmids using Lipofectamine 3000. After 48 hours, cells were lysed in Passive Lysis Buffer and processed further. By using the Dual-Luciferase Reporter Assay System (Promega) following the manufacturer’s instructions, the luciferase activity was measured by luminometry, and relative light units were normalized by Renilla luciferase.

### 2.9 Immunofluorescence

MIN6 cells were seeded into a 12-well plate (5 × 10^^5^ per well) with a microscope cover glass (Solarbio, China) one day prior to transfection. Cells were cotransfected with circ006029 or NC siRNA and the mCherry-GFP-LC3b plasmid. Forty-eight hours posttransfection, the cells were treated with or without 100 nM Rapamycin (Abiowell, China) for 10 hours. Then, the cells were fixed with 4% paraformaldehyde, and LC3b fluorescence was examined with a fluorescence microscope (KEYENCE BZ-X800, China) using a 60x oil-immersion objective.

### 2.10 Immunoblot Analysis

An immunoblot was conducted to measure corresponding protein expression. The MIN6 cells were washed gently with PBS and lysed with NP-40 (Beyotime Biotechnology, Shanghai, China) on ice for 20 minutes. All samples were centrifuged at 4°C for 20 minutes at 12000xg. The supernatant was collected into the new Eppendorf tube, mixed well with 5× loading buffer (NCM Biotech, China), and then boiled at 97°C for 5 minutes. Lysates were electrophoresed by SDS-PAGE and transferred to a PVDF membrane. After 5% bovine serum albumin (BSA, Biosharp, China) blocking and TBS-T washing at room temperature, membranes were incubated with primary antibodies and species-specific secondary antibodies. GAPDH and β-tubulin were employed as internal controls. The following antibodies were used: CCAR1 (Affinity; cat #DF2281), PCNA (Affinity; cat #AF0239), Cyclin D2 (Affinity; cat #AF5410), Cleaved-caspase-3 (Cell Signaling Technology; cat #9661), BAX (Proteintech; cat #50599-2-Ig), P62 (Abcam; cat #AB109012), LC3 (Proteintech; cat #14600-1-AP), p-mTOR (Proteintech; cat #67778-1-Ig), mTOR (Proteintech; cat # 66888-1-Ig), p-AKT (Cell Signaling Technology; cat #4058S), AKT (Cell Signaling Technology; cat #4685), Bcl2 (Affinity; cat #AF6139), iNOS (Proteintech; cat #18985-1-AP), p-P65 (Proteintech, cat #82335-1-RR), P65 (Proteintech, cat #10745-1-AP), GAPDH (ABclonal; cat #AC002), and β-tubulin (Proteintech; cat #10094-1-AP).

### 2.11 Differentially Expressed mRNA Sequencing and Clustering Analysis

The MIN6 cells were transfected with circ006029 or NC siRNA. After 48 hours, the total RNA was extracted from the cells by using TRIzol reagents (Thermo Fisher Scientific). RNA integrity was assessed using an Agilent 2100 Bioanalyzer (Agilent Technologies, Santa Clara, CA, USA), and RNA quantity and quality were determined using a NanoDrop 2000. The mRNA sequencing was performed on an Illumina Novaseq 6000 platform. *p* value < 0.05 and fold change > 2 or fold change < 0.5 were set as the threshold for significantly Differentially Expressed Genes (DEGs). The normalized expression level of each group was further analyzed with Hierarchical Clustering (HCL), and data were presented by using TreeView software (v. 1.5). The color blue represents low expression, while red represents high expression. In addition, the differentially expressed mRNAs from sequencing data were applied for GO and KEGG analysis.

### 2.12 Statistical Analysis

GraphPad Prism 9.0 software was used for data analysis. All data were expressed as mean ± Standard Deviation (mean ± SD). A t-test was conducted to compare differences among groups. Difference with *p* < 0.05 was considered statistically significant.

## 3 RESULTS

### 3.1 circ006029 is Increased in Cytokine-induced Injured MIN6 Cells

In our previous study, to identify the potential functions of circRNAs at the initial stage of T1DM, we treated MIN6 cells with a cytokine cocktail containing IFN-γ, IL-1β, and TNF-α and preliminarily screened dysregulated circRNAs using RNA microarrays. In the present study, our experimental results were in line with previous findings [15], demonstrating upregulation of circ006029 in MIN6 cells undergoing cytokine-induced inflammatory injury (Fig. **1A**; statistics in Supplementary Fig. **S1**). Circ006029 (ID: mmu_circ_0000172; Position: chr10:62233602-62253790) belongs to exonic circRNA and is derived from the exons of CCAR1. circBase shows the specific junction site of circ006029 (Fig. **1B**), and thus we designed specific primers for both circ006029 and CCAR1. Furthermore, qRT-PCR showed that circ006029 exhibited a more stable characteristic than its linear counterpart, CCAR1, under RNase R digestion or Actinomycin D interference (Figs. **1C** and **D**). For the localization of circRNA, RNA fractionation assays were further performed. The cytoplasmic and nuclear RNA of MIN6 cells were separated and extracted, and qRT-PCR was performed to determine circ006029 expression in the cytoplasm and nucleus. Here, the expression level of circ006029 was observed to be slightly higher in the cytoplasm than in the nucleus (Fig. **1E**). In brief, our preliminary data verified the increase of circ006029 in cytokine-induced damaged MIN6 cells and the stability of circ006029, revealing a potential role that circ006029 might serve in inflammatory cytokine-induced damaged pancreatic β-cells.

### 3.2 Knockdown of circ006029 Facilitates Proliferation and Alleviates Injury of MIN6 Pancreatic β-cells

Owing to the upregulation of circ006029 in cytokine-induced pancreatic β-cell damage, we speculated that circ006029 might play a detrimental regulatory role in β-cell destruction induced by inflammatory cytokines. Firstly, we verified the specific knockdown efficiency of circ006029 siRNA in MIN6 cells transfected with circ006029 or NC siRNA. As shown in Figs. (**2A**, **B**), the expression level of circ006029 was significantly decreased in MIN6 cells with circ006029 knockdown, while the mRNA and protein levels of CCAR1 displayed no significant change in the group with circ006029 knockdown (Figs. **2A**, **B**, Statistics in Supplementary Fig. **S2**). CCK-8 assay revealed that the inhibition of circ006029 could promote the proliferation of MIN6 β-cells (Fig. **2C**). Meanwhile, circ006029 inhibition could increase the protein levels of PCNA and cyclin D2 (Fig. **2D**, Statistics in Supplementary Fig. **S3**). Next, we observed the regulatory effect of circ006029 on cytokine-induced β cell damage. The flow cytometry data manifested a decreased apoptosis rate (Q2+Q3) in MIN6 cells with circ006029 knockdown, and the necrosis rate (Q1) was concurrently reduced compared with the cells transfected with NC siRNA (Fig. **2E**). The expression levels of apoptosis-related proteins, cleaved-caspase 3 and bax were observed to be declined in MIN6 cells with circ006029 knockdown, (Fig. **2F**, Statistics in Supplementary Fig. **S4**). These data demonstrated an important role of circ006029 in regulating β-cell proliferation and apoptosis.

### 3.3 Knockdown of circ006029 Increases MIN6 Cell Autophagy Through the AKT/mTOR Signaling Pathway

Autophagy is a protective mechanism maintaining cellular homeostasis and may protect cells from apoptosis [5]. Thus, we sought to explore whether circ006029 could improve β-cell survival through regulating β-cell autophagy. Preliminary immunoblotting results displayed a significant downregulation of p62, an increased ratio of LC3 II/I, and the upregulated levels of ULK and Beclin 1 in MIN6 cells with circ006029 inhibition, giving a hint of the participation of circ006029 in β-cell autophagy (Fig. **3A**, Statistics in Supplementary Fig. **S5**). Autophagy flux was monitored in MIN6 cells transfected with GFP-mCherry-LC3b plasmids to measure tandem fluorescence under different conditions. In a standard autophagy flux assay, GFP-mCherry-LC3b shows green (due to GFP) and red (due to mCherry) double fluorescence in Autophagic Vesicles (AVs) and only red fluorescence in Autolysosomes (ALs), indicating the spatiotemporal progress of formation and maturation of AVs. As shown in Fig. (**3B**), the increased autophagic flux was observed to occur in MIN6 cells transfected with circ006029 siRNA. The cells with circ006029 knockdown showed more apparent, brighter red granular AVs and ALs than the control. Such observed results were more evident when cells were treated with Rapamycin, an autophagy inducer that promotes autophagy through inhibition of the nutrient-sensing molecule mammalian Target of Rapamycin (mTOR) [18, 19] (Fig. **3B**, Statistics in Supplementary Fig. **S6**).

Based on these findings, we further confirmed whether circ006029 regulates β-cell autophagy through the AKT/mTOR pathway. As shown in Fig. (**3C**) (Statistics in Supplementary Fig. **S7**), the decreased phosphorylation of mTOR (p-mTOR) suggested an activated autophagy process in MIN6 cells with circ006029 knockdown. Then, we applied Rapamycin to observe the effect of circ006029 on autophagy activation. Decreased expression of p-mTOR implied enhanced autophagy in MIN6 cells with circ006029 knockdown. Furthermore, lower levels of p62 and an increased LC3II/I ratio were observed in cells transfected with circ006029 knockdown, with or without rapamycin treatment (Fig. **3D**; statistics in Supplementary Fig. **S8**). As illustrated in Fig. (**3C**), the decreased phosphorylated AKT (p-AKT) suggested that AKT participates in circ006029-regulated MIN6 cellular events as well. Thus, we further employed the pan-AKT kinase inhibitor Capivasertib (AZD5363) to determine whether AKT inhibition affected circ006029 regulation on β-cell autophagy. Capivasertib is a novel pyrrolopyrimidine-derived compound that can inhibit three isoforms, AKT1, AKT2, and AKT3, preventing the phosphorylation of the AKT downstream substrates and resulting in an accumulated p-AKT level and an inhibited AKT kinase activity [20]. Immunoblotting results revealed that the levels of p-AKT, p-mTOR, and p62 were reduced in MIN6 transfected with circ006029 siRNA, while the ratio of LC3 II/I was increased (Fig. **3E**, Statistics in Supplementary Fig. **S9**), whether with the application of Capivasertib or not. The autophagy level was further augmented after using Capivasertib. These data suggested that circ006029 participated in autophagy regulation *via* the AKT/mTOR pathway.

### 3.4 Knockdown of circ006029 Increases Autophagy and Concomitantly Attenuates Apoptosis of MIN6 Cells

To investigate whether altered autophagy affects MIN6 cell apoptosis, we further measured the expression of apoptosis-related proteins, such as Bcl-2, Bax, and cleaved caspase-3, after treating cells with Rapamycin or Capivasertib, respectively. We observed reduced apoptosis in MIN6 cells with circ006029 knockdown compared to cells transfected with NC siRNA, regardless of Rapamycin or Capivasertib treatment. The dampened apoptosis process was supported by reduced expression of Bax and cleaved caspase-3 (Fig. **4A**, **B**; statistics in Supplementary Fig. **S10** and **S11**). To summarize, the data above underscore that inhibition of circ006029 increases autophagy and, concomitantly, attenuates apoptosis in MIN6 cells *via* AKT/mTOR regulation.

### 3.5 Circ006029 May be Involved in the Inflammatory Response of β-cells

Given the increase in circ006029 in β-cells treated with inflammatory cytokines, we hypothesized that circ006029 may also be involved in inflammatory pathways, such as NF-κB, an essential transcriptional factor that contributes to inflammation and the immune response [21]. Co-transfection of the NF-κB activity luciferase reporter plasmid and circ006029 siRNA could result in a significantly decreased activity of NF-κB (Fig. **5A**). The following immunoblotting results also underscored the inactivation of the NF-κB signaling, which displayed a reduced level of iNOS and p-p65 (Fig. **5B**, Statistics in Supplementary Fig. **S12**). These results imply that circ006029 may play a regulatory role in pancreatic islet inflammation.

### 3.6 Changed Expression Profile of mRNAs in circ006029-depleted MIN6 Cells

To investigate the potential mechanisms by which circ006029 participates in inflammatory cytokine-induced β-cell destruction, transcriptome sequencing was conducted to identify differentially expressed protein-coding genes under circ006029 knockdown. Hierarchical cluster analysis manifested 73 upregulated mRNAs and 59 downregulated mRNAs in terms of the criteria of fold change (FC) > |2.0| and *p* < 0.05, upon the knockdown of circ006029 (Figs. **6A**, **B**). We selected 4 mRNAs (Lgals3, Pax4, Spink1, Ifitm10) from the sequencing data and validated their expression in MIN6 cells transfected with circ006029 siRNA. qRT-PCR results showed general consistency with transcriptome sequencing: depleting circ006029 in MIN6 cells significantly altered the expression of Lgals3, Spink1, and Pax4, but did not affect the expression of Ifitm10 (Fig. **6C**).

To investigate the potential biological interplay among these mRNAs, we performed GO analysis across three categories: Biological Process, Cellular Component, and Molecular Function. Figs. (**7A**, **B**) displayed significant enrichment of putative gene clusters in the top 30 upregulated and downregulated GO Terms. Among these GO Terms, immune- or cell viability-related terms accounted for a notable portion; in particular, apoptosis- and autophagy-regulatory genes were observed in these enrichments. To illustrate, pro-apoptosis-related genes cytochrome c, testis (Cyct) and serine/threonine/tyrosine interacting-like 1 (Styxl1) were significantly downregulated after the knockdown of circ006029; Nucleotide-binding oligomerization domain containing 2 (Nod2), substantiated to protect cells from bacteria attacking through promoting autophagy in Crohn diseases [22], and Galectin-3 (Lgals3), verified to interact with autophagy-related proteins and facilitate autophagy [23], were both displayed an increased expression in circ006029-knockdown β cells. Additionally, KEGG pathway analysis showed that the differentially up- or down-regulated genes were mainly enriched in the immune system, endocrine and metabolic disease, and signal transduction pathways (Figs. **7C**, **D**). These pathways likewise involved genes that were shown to regulate apoptosis, such as lymphocyte-specific 1 (Lsp1) [24]. Thus, these data imply that circ006029 may play a regulatory role in pancreatic β-cell injury.

To bring it all together, our investigation identified a regulatory role of circ006029 in pancreatic β-cells. Knockdown of circ006029 alleviates β-cell apoptosis and improves cell proliferation by promoting cell autophagy *via* the AKT/mTOR signaling pathway. It also suggests that circ006029 could be a potential intervening target during early pancreatic cell injury, due to its ability to attenuate cell damage and restore pancreatic cell function.

## 4 DISCUSSION

Type 1 Diabetes Mellitus (T1DM) is characterized by autoimmune-mediated destruction of pancreatic β cells, where chronic insulitis and subsequent infiltration of proinflammatory cytokines (*e.g.*, IFN-γ, IL-1β, TNF-α) released by falsely activated T cells are central causes of β-cell dysfunction and loss [3, 25]. Although advanced treatments have been developed to manage T1DM [26, 27], the molecular cascades or corresponding cell-injury mechanisms governing β-cell vulnerability during early immune attack in T1DM remain incompletely defined. Elucidating these pathways is still crucial for developing strategies to preserve β-cell mass and function in preclinical stages of the disease.

Non-coding RNAs (ncRNAs) have emerged as key regulators of β-cell cellular life activities in T1DM. Among them, lncRNAs and miRNAs have been shown to play essential roles in modulating inflammation, apoptosis, and metabolic stress responses in pancreatic β cells [12, 28-30]. Another class of ncRNAs, circular RNAs (circRNAs), has recently gained attention for their stable structure and context-specific regulatory functions in T1DM. Prior studies highlight their involvement in β-cell homeostasis. For example, circHIPK3 protects β-cells from apoptosis, whereas circPPM1F exacerbates injury by activating M1 macrophages. Such evidence underscored the necessity of exploring circRNAs as potential modulators of βcell injury in T1DM [13, 14].

Based on our preliminary studies [15, 17], we further investigated the possible functions of the increased circRNA, circ006029, in β-cell damage. In the present study, we yet again verified the upregulated circ006029 in mouse pancreatic β-cells (MIN6) upon the stimulation of cytokine cocktails of IFN-γ, IL-1β, and TNF-α, confirming the potential involvement of circ006029 in β-cell injury (Fig. **1A**). The knockdown of circ006029 was found to promote MIN6 proliferation, reduce the apoptotic ratio, and decrease the levels of apoptosis-related proteins (Fig. **2**). Thus, we speculated that the presence of circ006029 may detrimentally regulate β-cell survival.

Intriguingly, increased autophagy was observed in MIN6 cells with circ006029 knockdown. Autophagy is an important cellular process responsible for maintaining cellular homeostasis [5]. It is an important mechanism that protects β-cells from apoptosis upon the initiation of β-cell damage [31]. Moreover, increasing evidence indicates the importance and involvement of circRNAs in cell autophagy [32, 33]. Our data substantiated enhanced autophagy in circ006029-knockdown MIN6 cells (Fig. **3**), manifested as altered expression of autophagy-related proteins and altered fluorescence in Autophagic Vesicles (AVs) and Autolysosomes (ALs), respectively. As the evidence of ncRNA-involved cell autophagy *via* the AKT/mTOR pathway has been increasing [34, 35], we further traced potential associated molecules. Significant alterations in phosphorylated AKT/mTOR, apoptosis- and autophagy-related protein levels were observed in MIN6 cells with circ006029 knockdown. These data indicated that circ006029 regulation of β-cell survival might, in all likelihood, be associated with the AKT/mTOR signaling pathway (Fig. **3**). The AKT pathway has been shown to negatively regulate cell apoptosis [36]. Interestingly, we observed that inhibition of circ006029 decreased AKT phosphorylation and slightly increased the level of the anti-apoptotic protein Bcl-2, thereby reducing MIN6 cell apoptosis. We also observed that the use of Rapamycin and Capivasertib, in circ006029-knockdown β cells, results in a greater inhibition of apoptosis (Fig. **4**). As we applied compounds to magnify the regulatory role of circ006029, considering the possibility that certain circumstances, such as metabolic changes or bypass regulation-induced protein regulation [37], might explain the non-significant or inconsistent (with *vs*. without drugs) change in the bcl-2 level, we thus speculated that this might explain the non-significant or inconsistent (with *vs*. without drugs) change in the bcl-2 level. Nevertheless, the protective role (*i.e.*, the promotion of autophagy and the inhibition of apoptosis) of circ006029 knockdown on MIN6 β-cells was still unignorable.

Considering that β-cell damage was mainly mediated by proinflammatory factors, we additionally explored NF-κB, a critical transcription factor known to be significant in the inflammatory response [21], to determine whether circ006029 participates in the β-cell inflammatory response. We observed significant inhibition of NF-κB activity and decreased protein levels of iNOS and p-p65 in β-cells with circ006029 knockdown (Fig. **5**), further elucidating a potential role of circ006029 in β-cell inflammation.

Additionally, in this study, a series of differentially expressed mRNAs was identified in β-cells with circ006029 knockdown, among which certain genes have recently been shown to contribute to cell autophagy, such as Nod2 and Lgals3 [22, 23]. Through Go analysis, we found that the top 30 upregulated and downregulated GO Terms focused on complement activation, immune responses, or cell viability-related activities. Through KEGG analysis, the pathways were mainly focused on the immune system, endocrine and metabolic diseases, and signal transduction, suggesting possible roles for circ006029 in regulating inflammatory responses and metabolic pathways, thereby contributing to pancreatic β-cell dysfunction and type 1 diabetes. The limitation of this study was the cell line application. While our study revealed circ006029's role in MIN6 cells, future work should include corresponding validation in primary human β-cells or INS-1 lines to assess regulatory potential.

## CONCLUSION

Our findings align with emerging evidence that circRNAs may modulate β-cell survival through metabolic pathways (*e.g.*, AKT/mTOR). However, we uniquely identified circ006029 as a negative regulator of autophagy in T1DM contexts. The promotion of βcell proliferation also substantiated the protective effects of circ006029 silence. Furthermore, we further proved that circ006029 might contribute to autophagy through the AKT/mTOR signaling pathway. All the results implied that the presence of circ006029 may drive a detrimental regulatory role in pancreatic β- cells. This may provide valuable evidence that circ006029 might be a potential target for alleviating β- cell damage in T1DM and rebuilding β-cell function.

## Figures and Tables

**Fig. (1):**
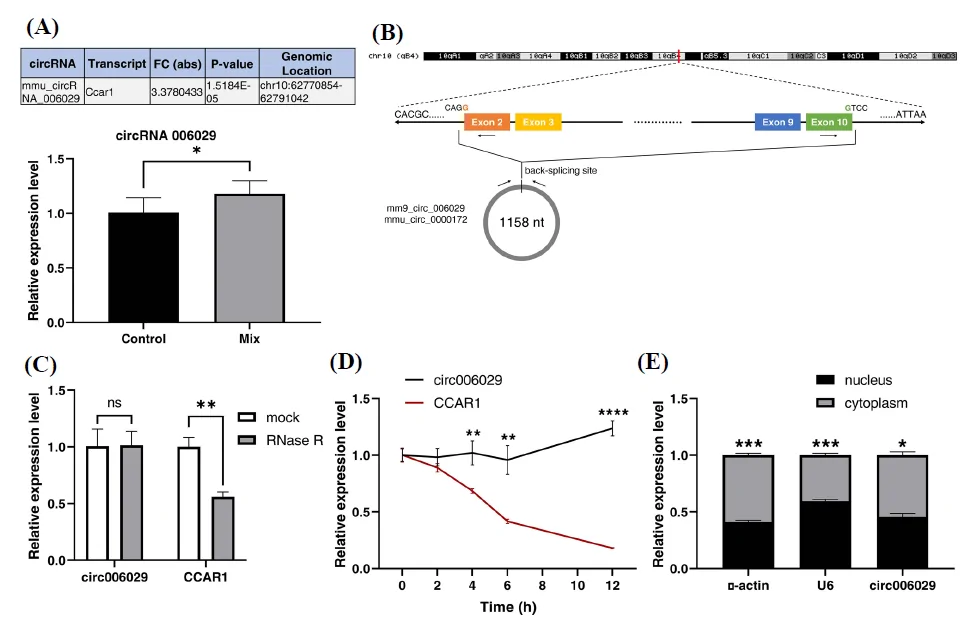
Characterization of circ006029 in β-cells. (**A**) circ006029 is upregulated in MIN6 cells treated with cytokine cocktails (shown as Mix) of 25 ng/ml IFN-γ, 2.5 ng/ml IL-1β, and 2.5 ng/ml TNF-α for 24 h (n=6). Relative expression level of circ006029 was measured by qRT-PCR. (**B**) Schematic illustration of circ006029 circularization. (**C**) The stability of circ006029 and its linear counterpart CCAR1 in MIN6 cells post-digested with RNase R (1 U per 3 ug RNA) (n=3). (**D**) The resistance of circ006029 and CCAR1 to actinomycin D (5 ug/ml) treatment. (**E**) The intracellular location of circ006029 in MIN6 cells was measured by subcellular separation assay (n=4). Data are presented as mean ± SD. *p ≤ 0.05, **p ≤ 0.01, ***p ≤ 0.001, ****p ≤ 0.0001, ns indicates no significance.

**Fig. (2):**
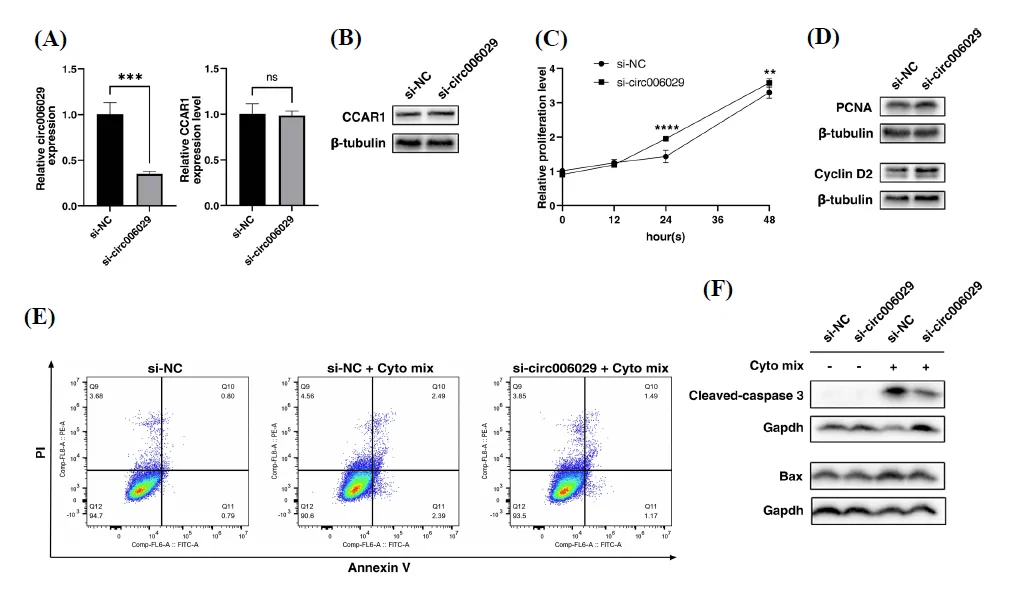
Knockdown of circ006029 promotes proliferation and attenuates β-cell injury. (**A** and **B**). The knockdown efficiency and specificity of transfection with circ006029 siRNA was examined by qRT-PCR, and the expression of CCAR1 was additionally measured by immunoblotting. (**C**) The relative proliferation level of MIN6 cells was determined by CCK-8 assay after the knockdown of circ006029. (**D**) The expression of proliferation-related proteins was upregulated in MIN6 cells transfected with circ006029 siRNA. (**E**). The apoptosis of MIN6 cells was measured by flow cytometry. Cells were transfected with NC and circ006029 siRNA, and treated with cytokine cocktails for 24 h, then cells were harvested and stained with Annexin V/PI. (**F**). Immunoblotting to detect apoptosis-related protein levels, includes cleaved-caspase 3 and bax (n=5). Data are presented as mean ± SD. *p ≤ 0.05, **p ≤ 0.01, ***p ≤ 0.001, ****p ≤ 0.0001, ns indicates no significance.

**Fig. (3):**
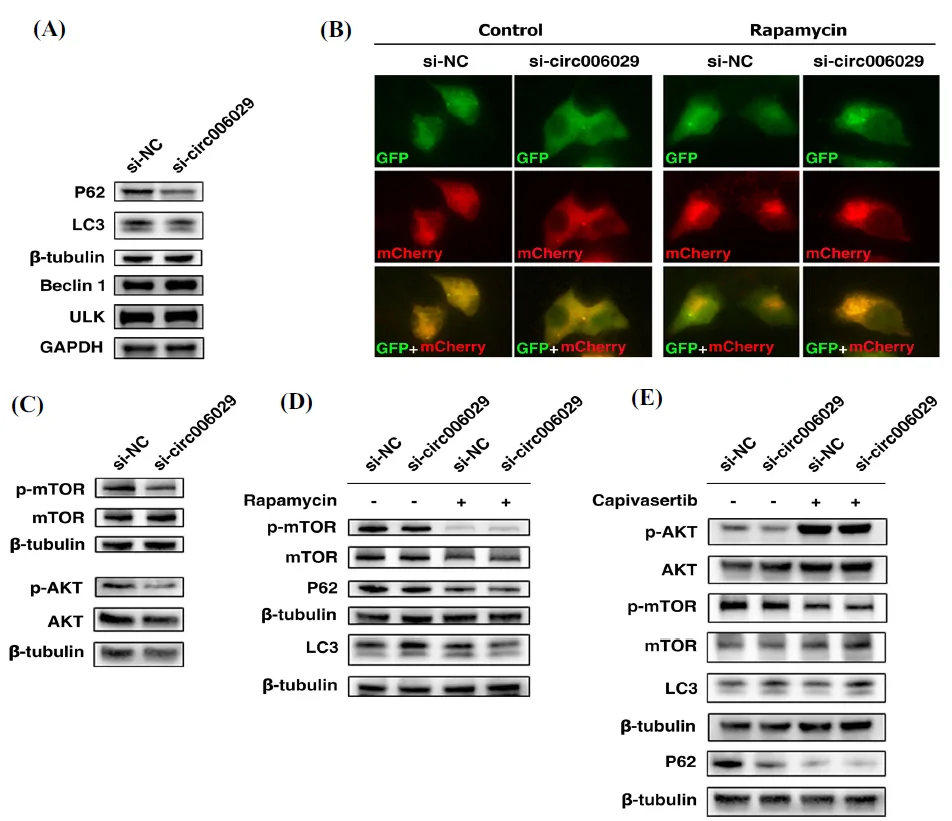
Knockdown of circ006029 increases β-cell autophagy through the AKT/mTOR signaling pathway. (**A**) Immunoblotting to detect autophagy-related protein p62 and LC3 after circ006029 knockdown of MIN6 cells. (**B**) MIN6 cells were co-transfected with NC and circ006029 siRNA and the mCherry-GFP-LC3b plasmid, then treated with Rapamycin (100 nM) for 16 h. GFP-LC3b and mCherry-LC3b were visualized using a confocal microscopy. (**C**) Phosphorylation of AKT (Ser473) and mTOR (Ser2448) were measured by immunoblotting after cells were transfected with NC and circ006029 siRNA. (**D**) Immunoblotting to detect autophagy-related proteins in MIN6 β-cells with circ006029 knockdown, followed by Rapamycin (100 nM) treatment for 16 h. (**E**) Immunoblotting to detect autophagy-related proteins in MIN6 cells with circ006029 knockdown, followed by Capivasertib (5 uM) treatment for 16 h. Data are presented as mean ± SD.

**Fig. (4):**
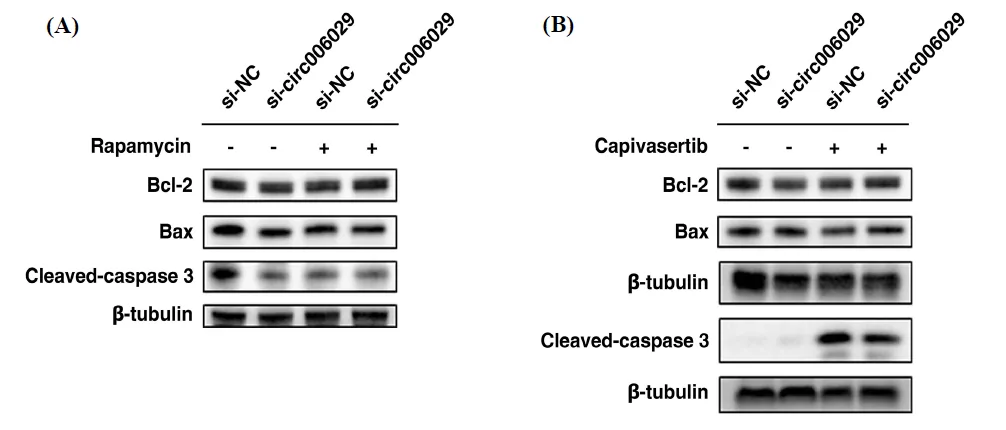
The autophagy participates in circ006029 regulating β-cell apoptosis. (**A**) Immunoblotting to detect apoptosis-related proteins in MIN6 β-cells with circ006029 knockdown, followed by Rapamycin (100 nM) treatment for 16 h. (**B**) Immunoblotting to detect apoptosis-related proteins in MIN6 β-cells with circ006029 knockdown, followed by Capivasertib (5 uM) treatment for 16 h. Data are presented as mean ± SD.

**Fig. (5):**
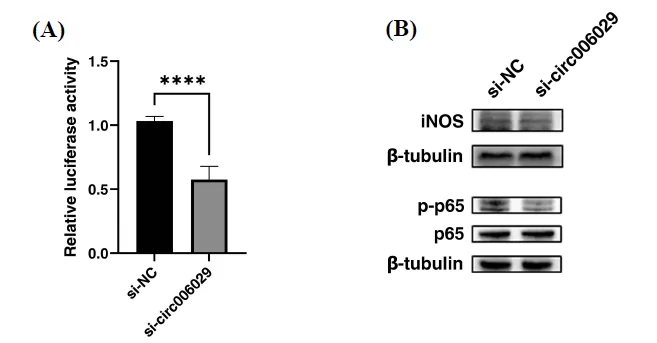
Circ006029 is participated in the inflammatory response of MIN6 β-cells. (**A**) Dual-Luciferase Reporter assay displayed the decrease of NF-κB activity in MIN6 cells with circ006029-knockdown. (**B**) Immunoblotting to detect iNOS and p-p65 expression level in MIN6 cells transfected with NC and circ006029 siRNA. Data are presented as mean ± SD. *p ≤ 0.05, **p ≤ 0.01, ***p ≤ 0.001, ****p ≤ 0.0001, ns indicates no significance.

**Fig. (6):**
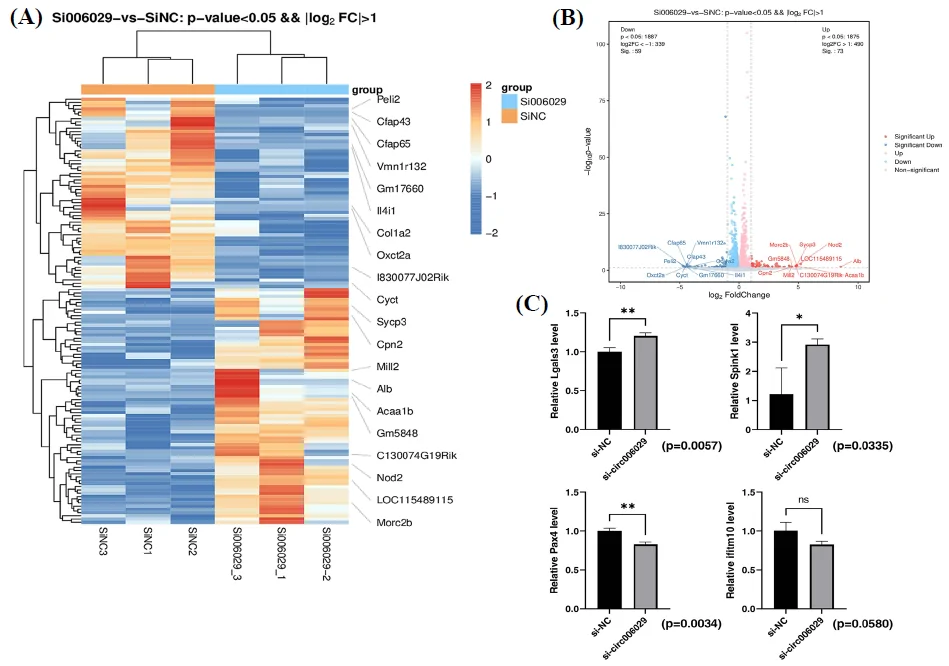
Result of the analysis for the differentially expressed mRNAs in MIN6 β-cells with circ006029-knockdown. (**A**) Heatmap of differential expressed mRNA profile in MIN6 cells with circ006029-knockdown versus negative control. (**B**) Volcano scatter of fold changes in mRNA expression in MIN6 cells with circ006029-knockdown versus negative control. (**C**) Validation of differentially expressed mRNAs associated with circ006029-knockdown was performed by qRT-PCR. Data are presented as mean ± SD. *p ≤ 0.05, **p ≤ 0.01, ns indicates no significance.

**Fig. (7):**
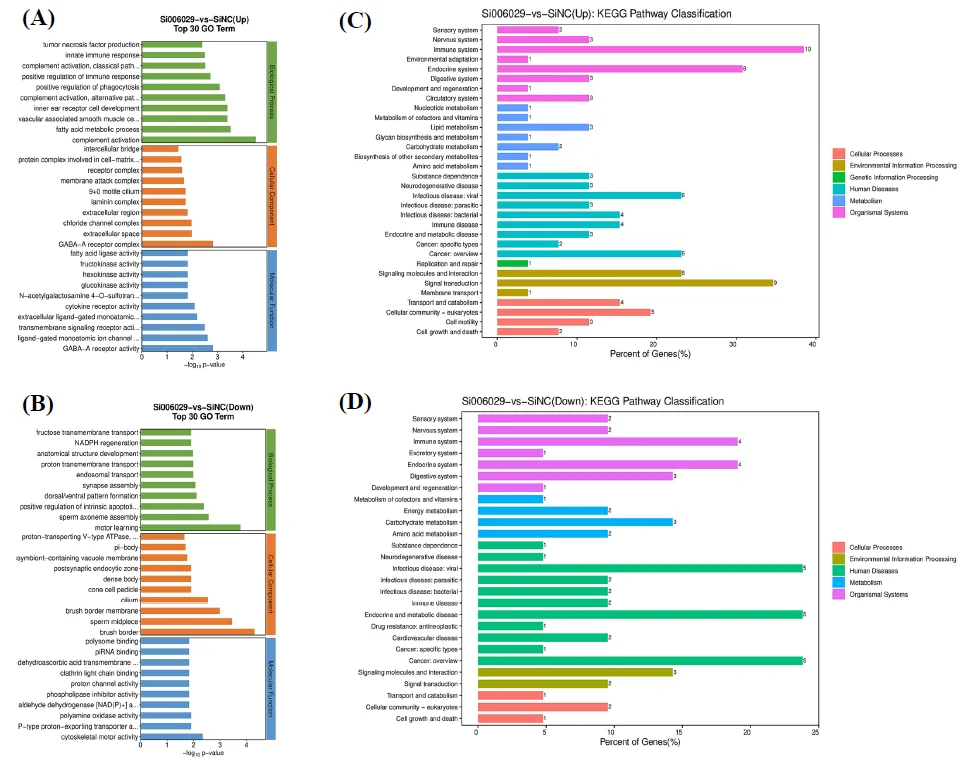
GO enrichment and KEGG pathway analysis of differentially expressed mRNAs in β-cells with circ006029-knockdown. (**A** and **B**) Histogram depicts the enriched GO terms associated with top 30 upregulated and top 30 downregulated mRNAs (*p* < 0.05). (**C** and **D**) Bar chart illustrates the top enriched KEGG pathways relevant to upregulated and downregulated mRNAs (*p* < 0.05).

## Data Availability

All data generated or analyzed during this study are included within the published article and are available from the corresponding authors upon reasonable request.

## LIST OF ABBREVIATIONS

T1DM: Type 1 Diabetes Mellitus
ncRNAs: Non-coding RNAs
lncRNAs: Long Non-coding RNAs
miRNAs: MicroRNAs
circRNAs: Circular RNAs
NC: Negative Control
DEGs: Differentially Expressed Genes
HCL: Hierarchical Clustering
