# Supplementary material for: Inhibition of Circular RNA 006029 Alleviates Pancreatic β-Cell Injury Through the AKT/mTOR Signaling Pathway
Source: Curr Med Chem. 2026 Feb 4;33(28):6073–86. doi: 10.2174/0109298673401334251201092718 (PMC13698028; doi:10.2174/0109298673401334251201092718)
Supplement: Supplementary file 1 [file CMC-33-28-6073_SD1.pdf]

Supplementary Material

Inhibition of Circular RNA 006029 Alleviates Pancreatic β-Cell Injury Through the AKT/mTOR Signaling Pathway

Zhen Wang[1,2], Zheli Luo[3], Li Wang[4], Jiaxing Feng[5], Kunlin Huang[5,*], Ying Zheng[5,*] and Zhiguang Zhou[2]

[1]Department of Metabolism and Endocrinology, Guilin Hospital of the Second Xiangya Hospital CSU, Guilin, 541100, China; [2]Key Laboratory of Diabetes Immunology, Department of Metabolism and Endocrinology, National Clinical Research Center for Endocrine and Metabolic Diseases, Ministry of Education, The Second Xiangya Hospital of Central South University, Changsha, 410011, China; [3]Department of Endocrinology, Shenzhen Second People's Hospital, First Affiliated Hospital of Shenzhen University, Shenzhen, 518000, China; [4]Department of Thoracic Surgery, The Second Xiangya Hospital, Central South University, Changsha, Hunan, 410011, China; [5]Center for Medical Research, The Second Xiangya Hospital, Central South University, Changsha, Hunan, 410011, China

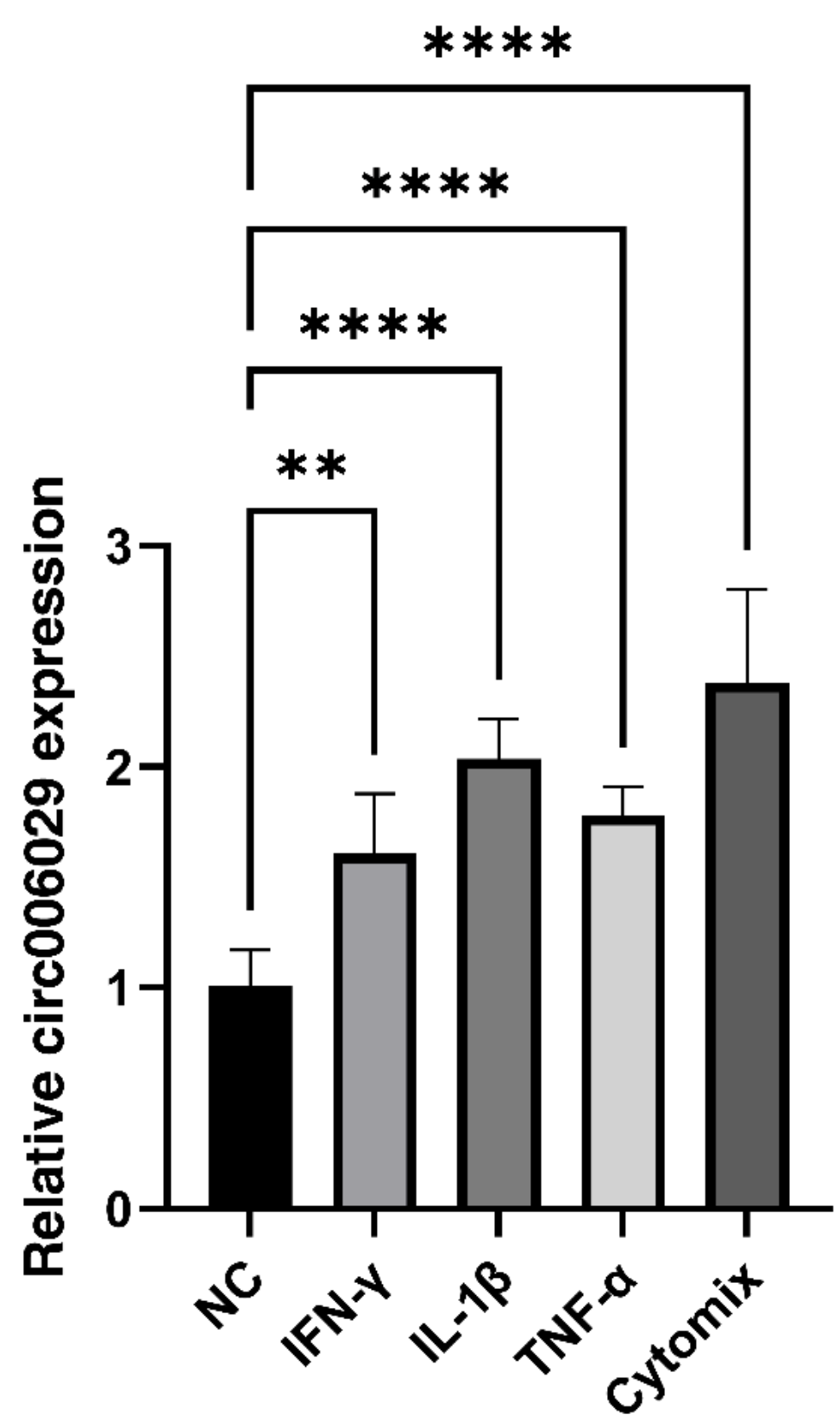

Fig. (S1). Cytokine-stimulated circ006029 expression. Statistics of Fig 1A of the manuscript.

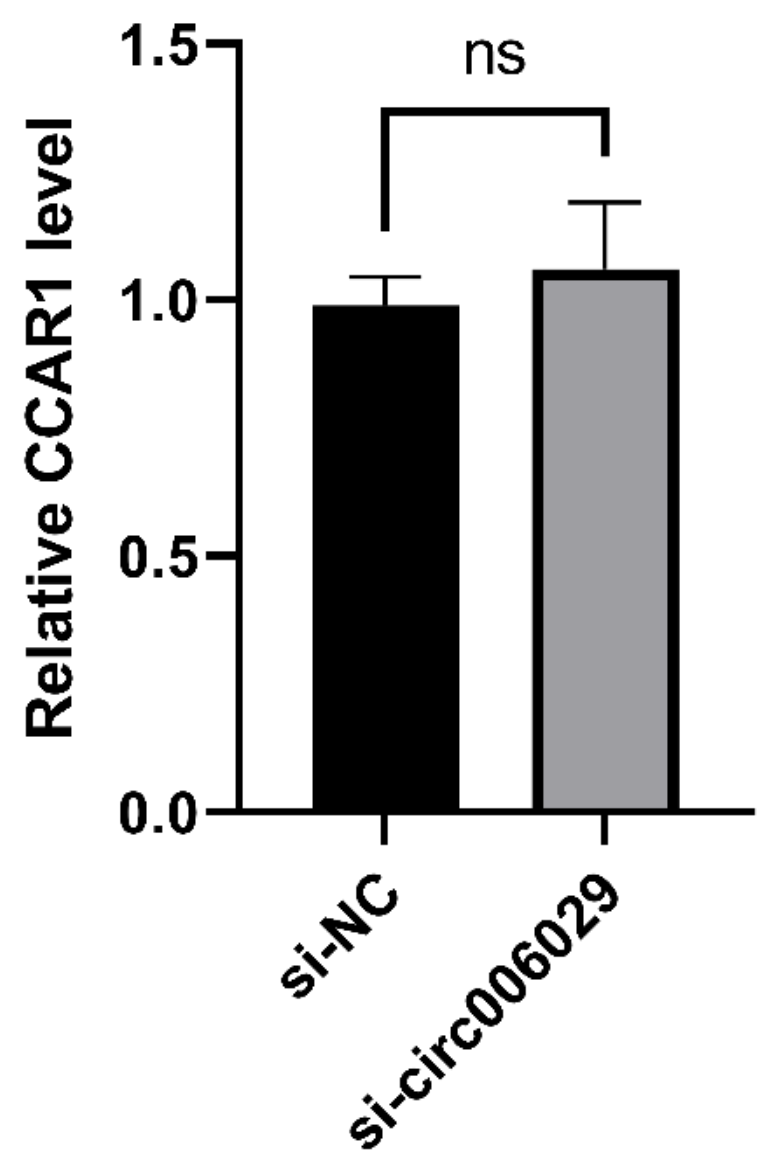

Fig. (S2). CCAR1. Statistics of Fig 2B of the manuscript.

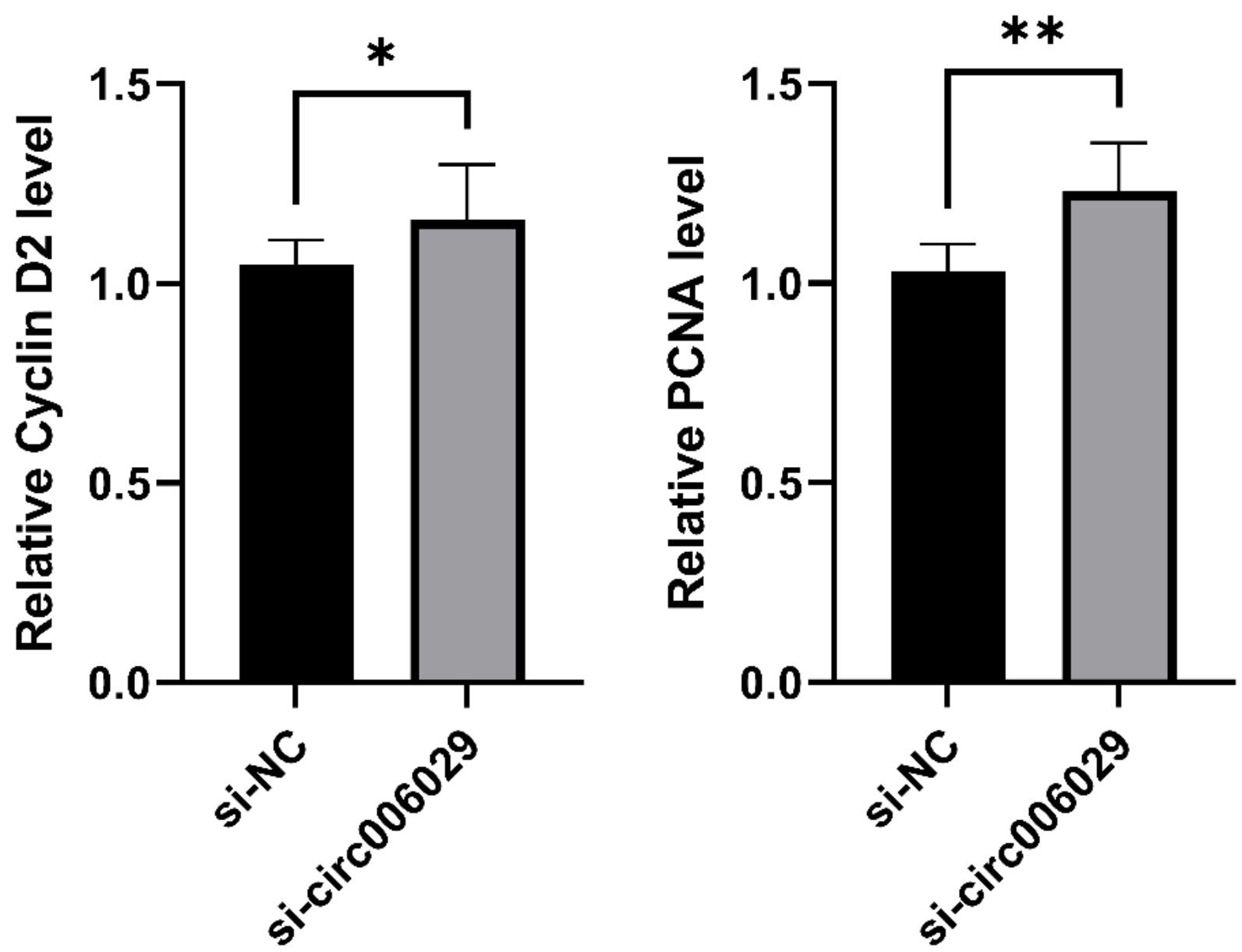

Fig. (S3). PCNA, Cyclin D2. Statistics of Fig 2D of the manuscript.

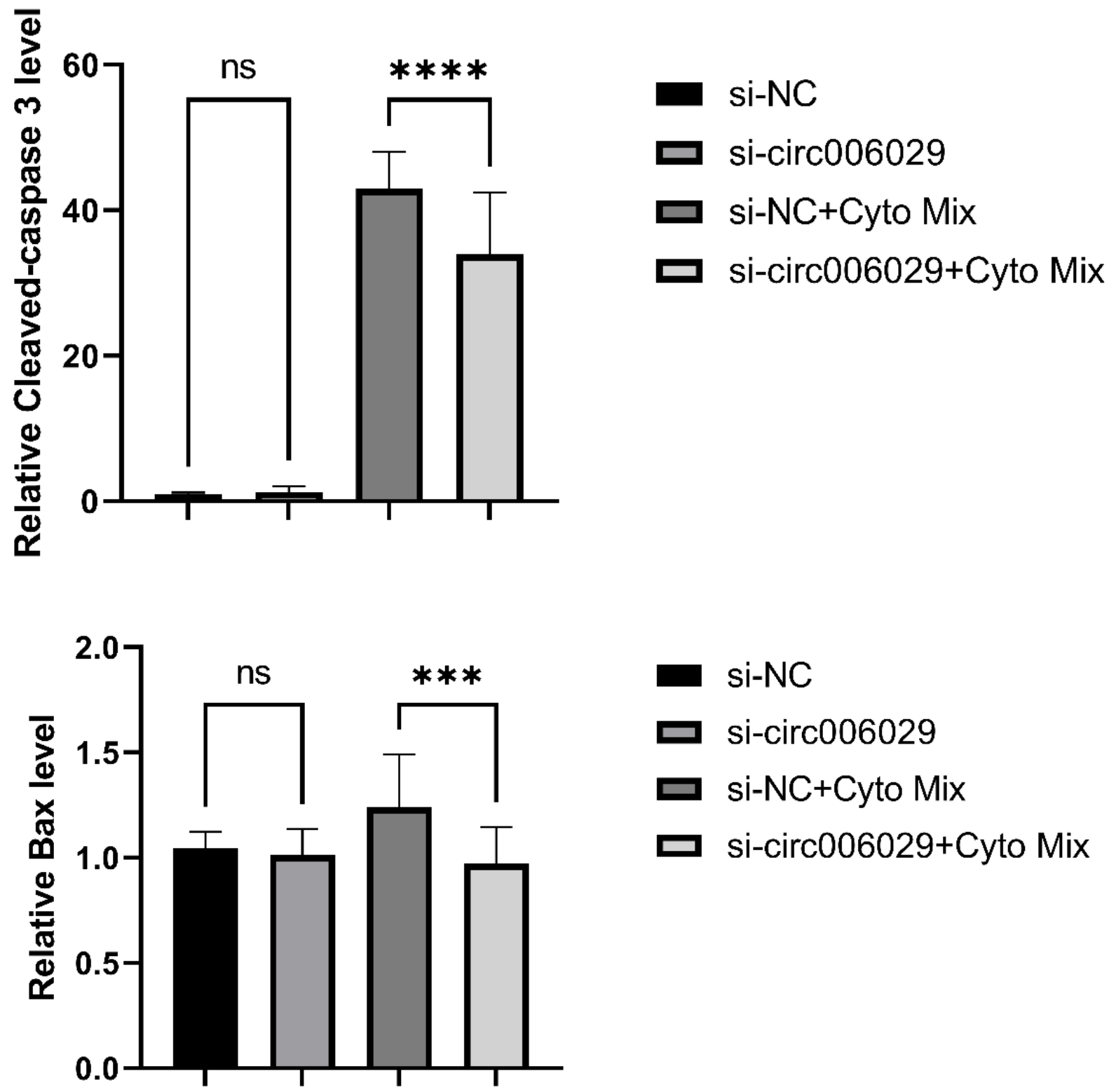

Fig. (S4). Cleaved-caspase 3, Bax. Statistics of Fig 2F of the manuscript.

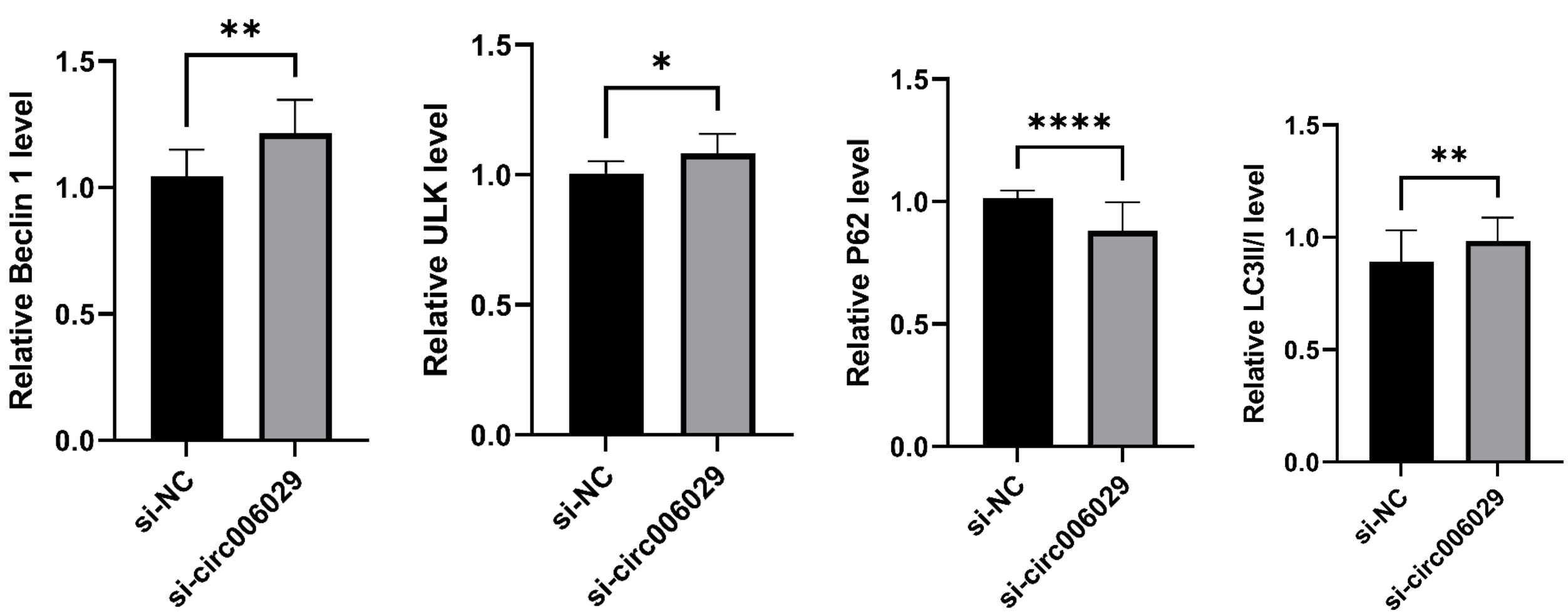

Fig. (S5). P62, LC3 II/I, Beclin 1, ULK. Statistics of Fig 3A of the manuscript.

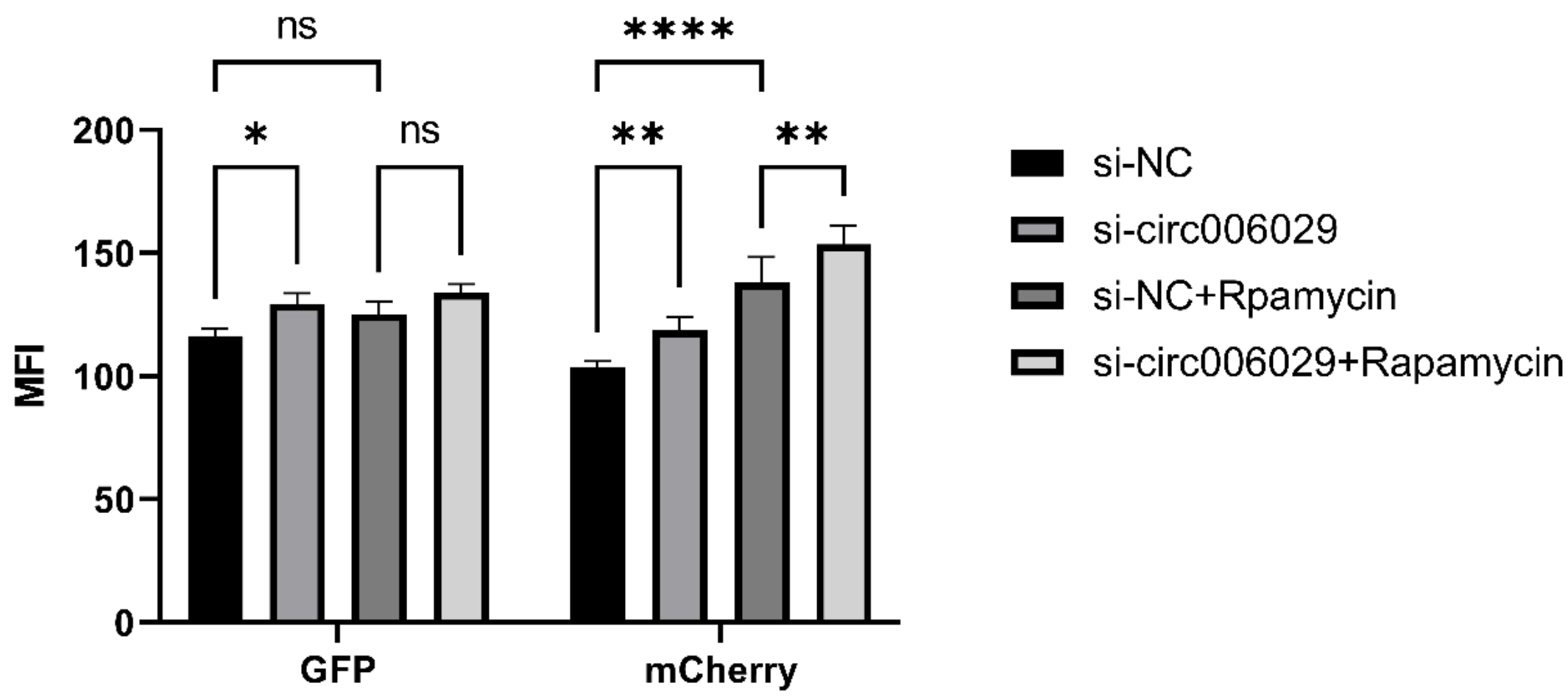

Fig. (S6). Immunofluorescence. Statistics of Fig 3B of the manuscript.

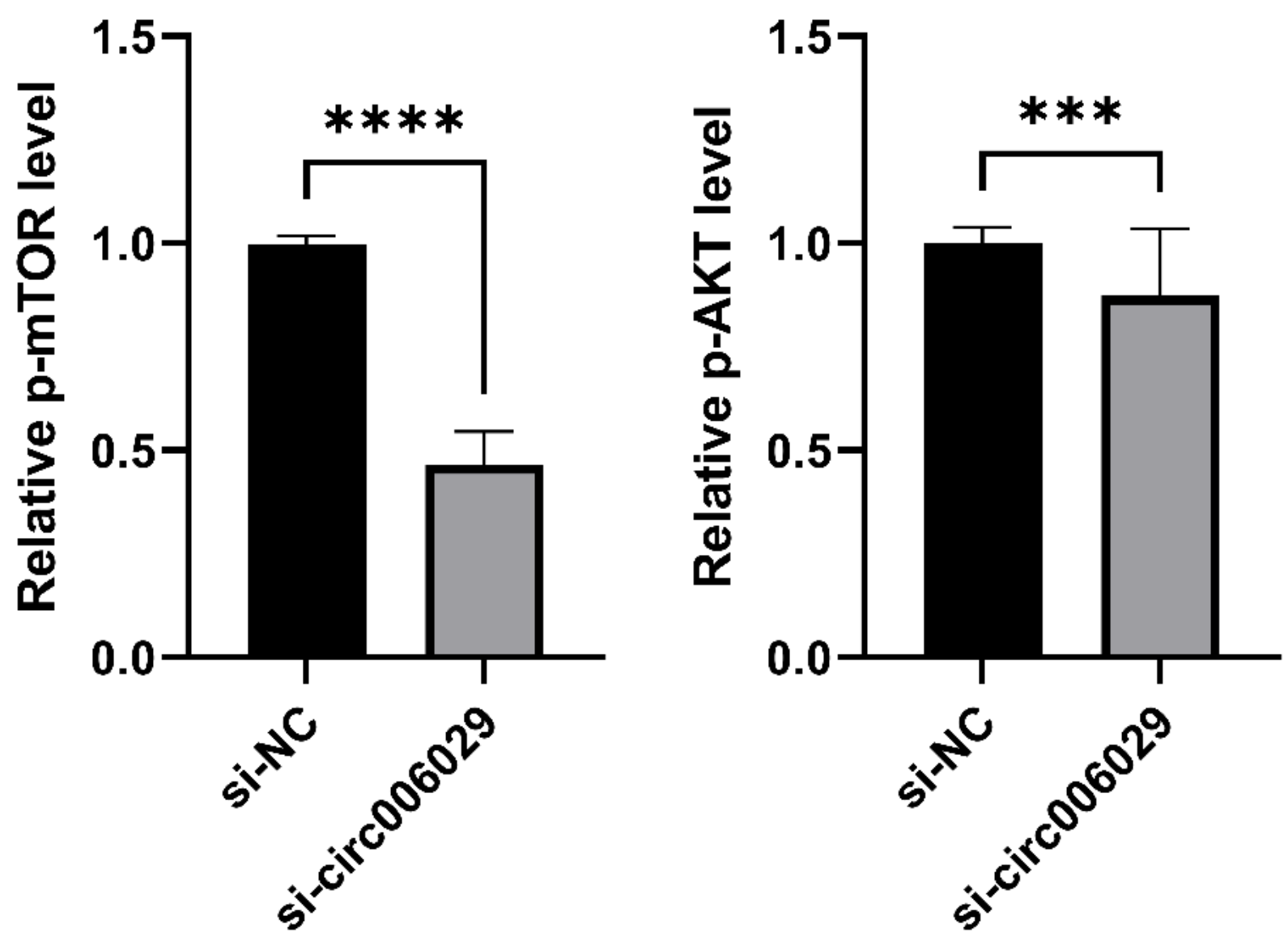

Fig. (S7). AKT, mTOR. Statistics of Fig 3C of the manuscript.

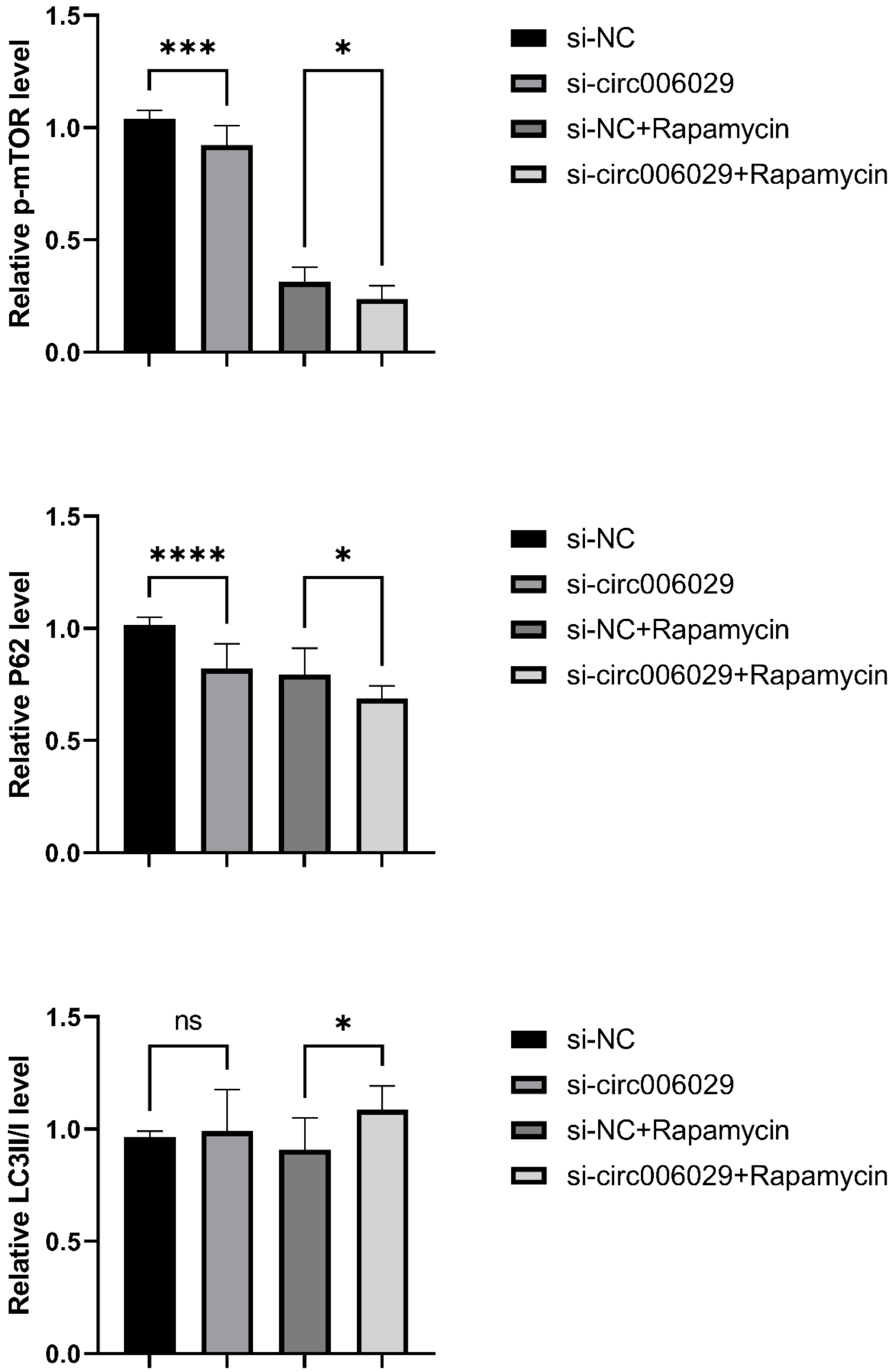

Fig. (S8). mTOR, P62, LC3 II/I. Statistics of Fig 3D of the manuscript.

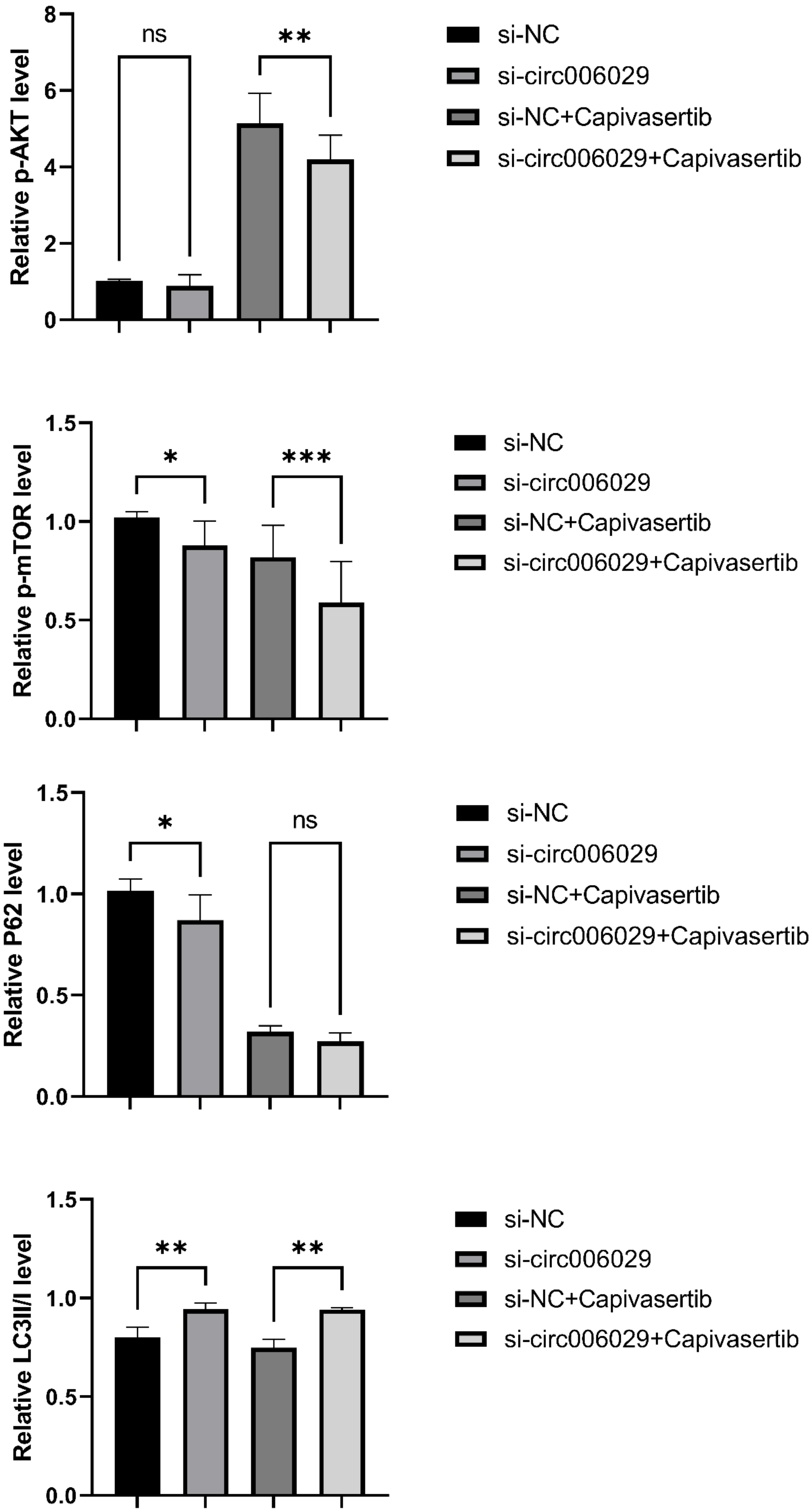

Fig. (S9). AKT, mTOR, P62, LC3 II/I. Statistics of Fig 3E of the manuscript.

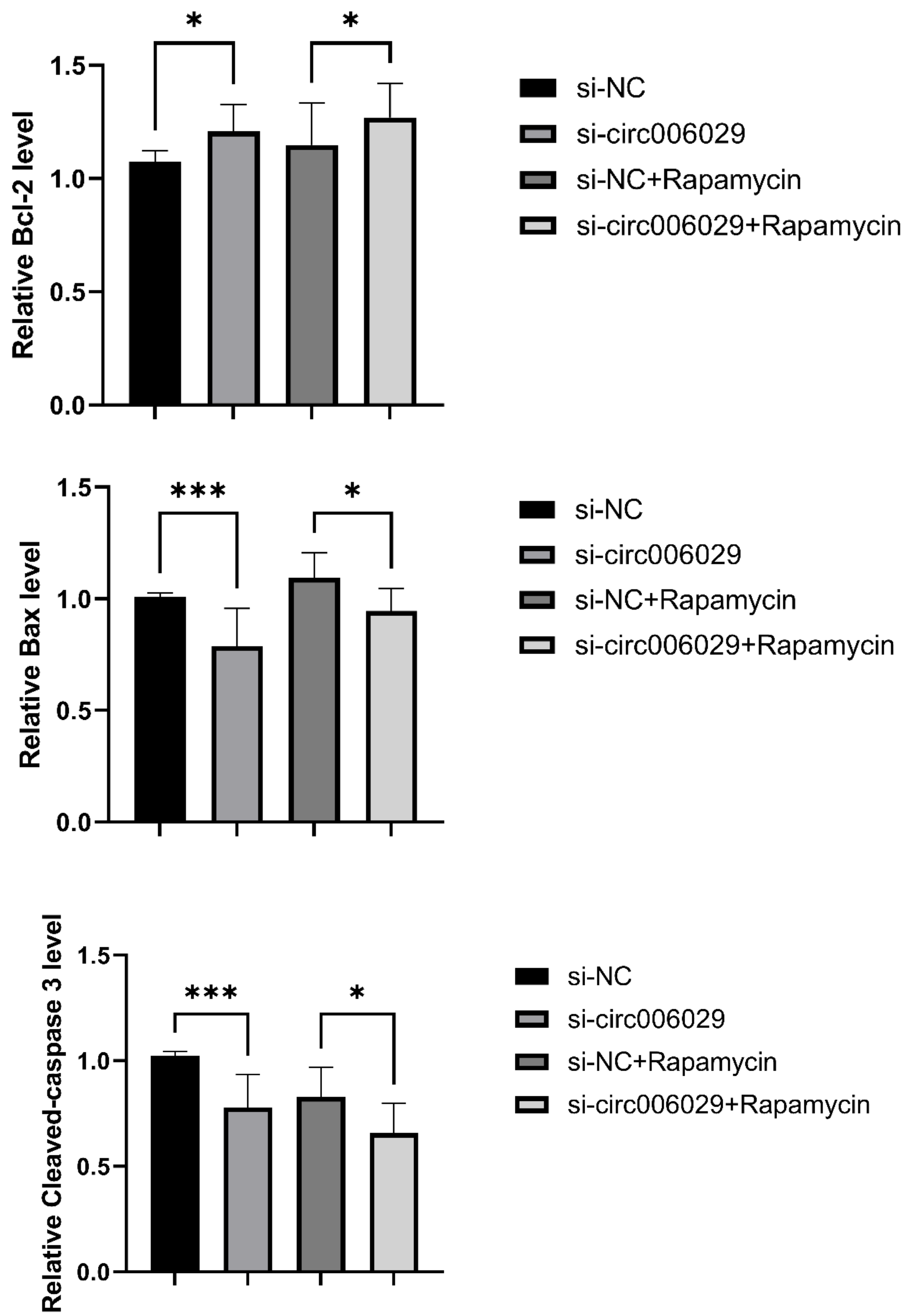

Fig. (S10). BCL2, BAX, Cleaved-caspase 3. Statistics of Fig 4A of the manuscript.

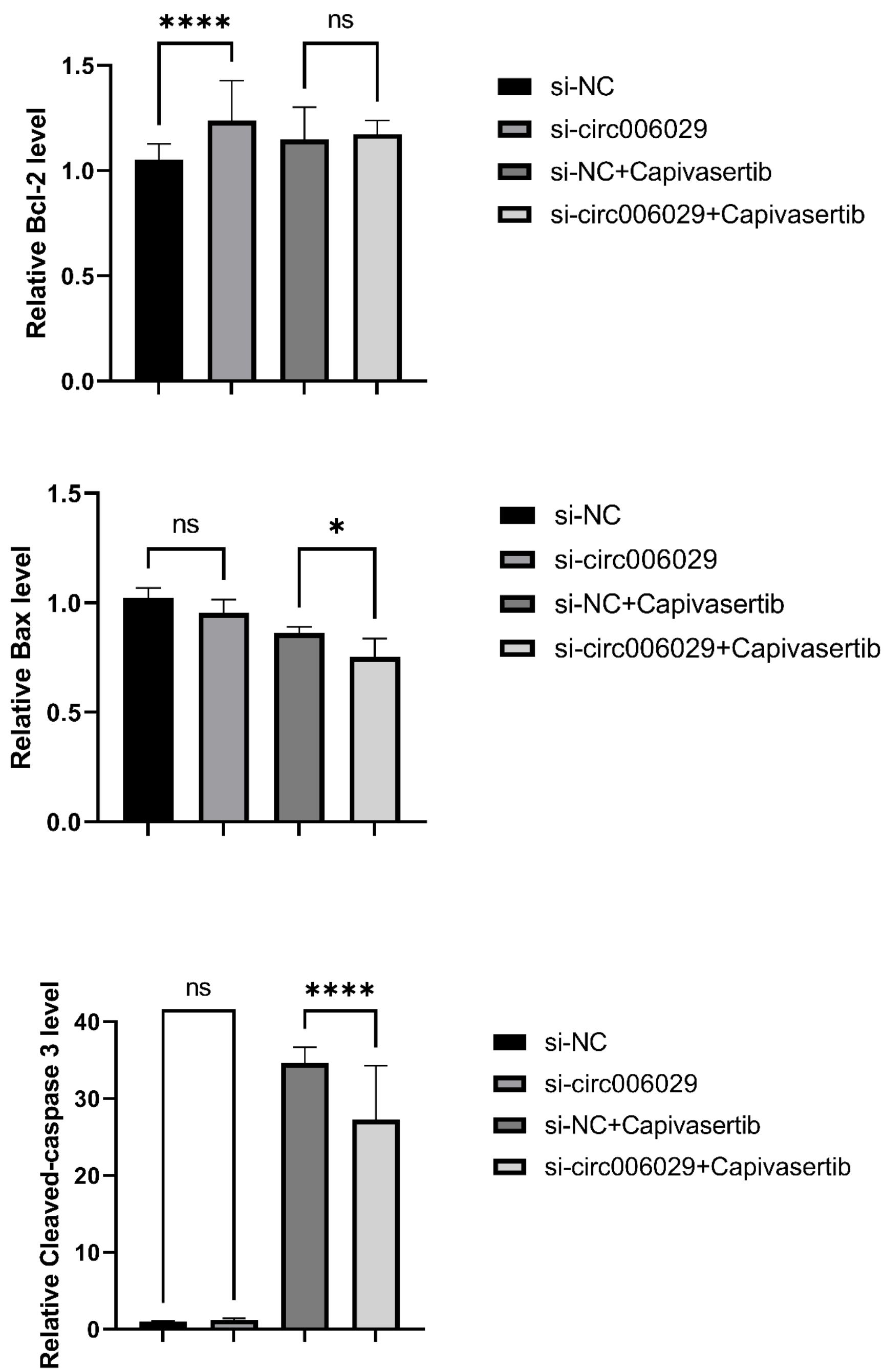

Fig. (S11). BCL2, BAX, Cleaved-caspase 3. Statistics of Fig 4B of the manuscript.

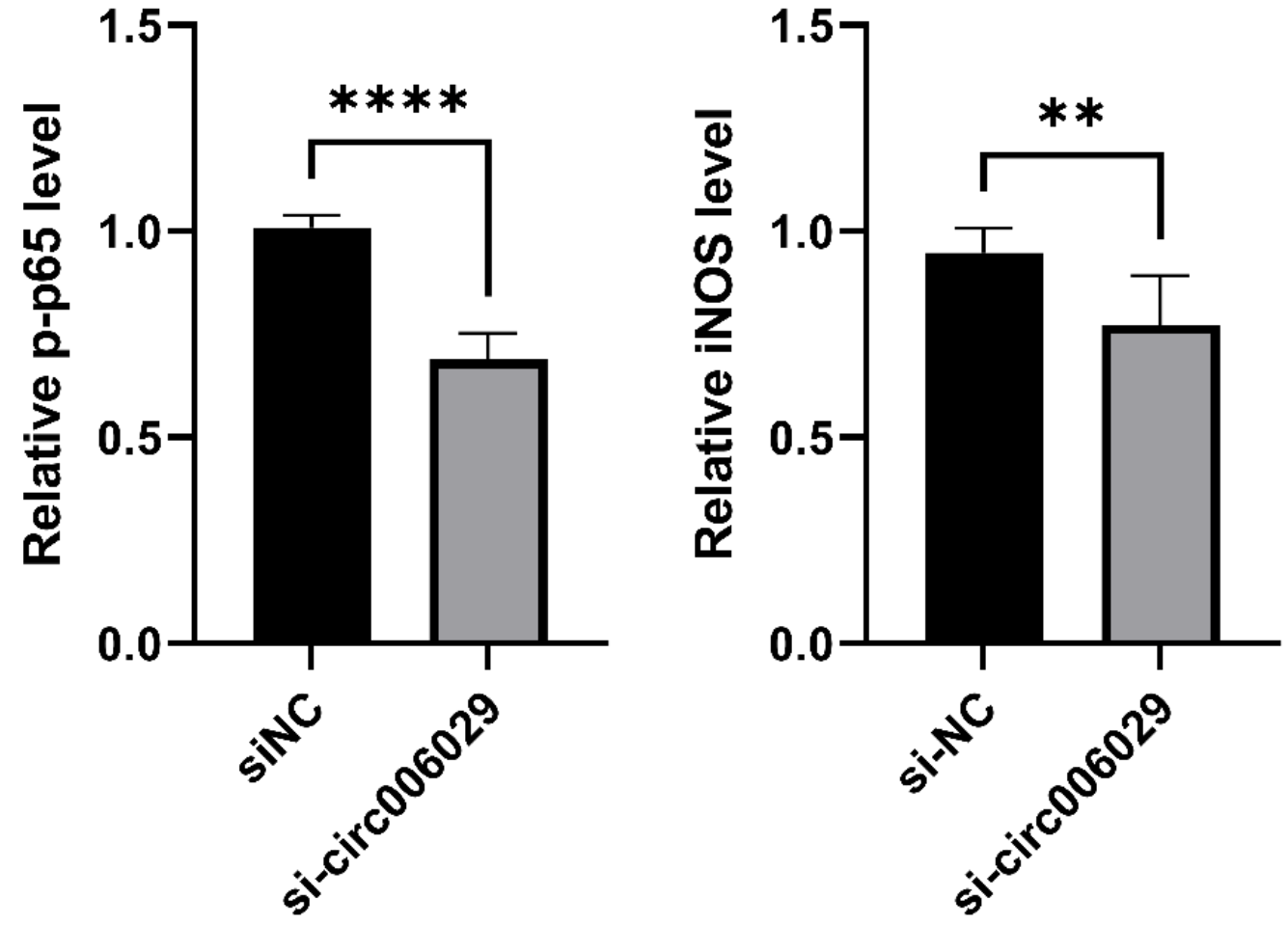

Fig. (S12). INOS, P65. Statistics of Fig 5B of the manuscript.

Table S1. Probe sequence for si-circRNA 006029.

Probe Name	Probe Sequence
si-circ006029-S	CUUUAGAUUGGUAUCUGCUTT
si-circ006029-AS	AGCAGAUACCAAUCUAAAGTT

Table S2. Primers for qPCR of CircRNA 006029.

Amplification primer name	Amplification primer sequence
006929-F	GAGAGTCCGTCGTGTCGTT
006029-R	AACTTTCACTTGTTTCCCTTCTA
CCAR1-F	ATCGCCACTCCTACCCATT
CCAR1-R	TTTAGTCCTTTGGAACTGAGAGC
Lgals3-F	AACCCAACGCAAACAGGATT
Lgals3-R	AGCGGGGGTTAAAGTGGAAG
Spink1-F	TGTGTGTGGGACTGACGGA
Spink1-R	AGGACAGGCTCTATGCGTTT
Pax4-F	TTGCCTGGGAGATCCAACAC
Pax4-R	GACTGGGAAGAACTGGAGCC
Ifitm10-F	AAGACGATGACGAACCCCAC
Ifitm10-R	AAGCCCAGGCAACAGAAGTT
U6-F	GGAACGATACAGAGAAGATTAGC
U6-R	TGGAACGCTTCACGAATTTGCG
β-actin-F	CGTGAAAAGATGACCCAGATCA
β-actin-R	TGGTACGACCAGAGGCATACAG
